# Machine Learning Algorithms to Classify and Quantify Multiple Behaviours in Dairy Calves Using a Sensor: Moving beyond Classification in Precision Livestock

**DOI:** 10.3390/s21010088

**Published:** 2020-12-25

**Authors:** Charles Carslake, Jorge A. Vázquez-Diosdado, Jasmeet Kaler

**Affiliations:** School of Veterinary Medicine and Science, Sutton Bonington Campus, University of Nottingham, Leicestershire LE12 5RD, UK; svzjv@exmail.nottingham.ac.uk (J.A.V.-D.); jasmeet.kaler@nottingham.ac.uk (J.K.)

**Keywords:** precision livestock farming, calves, sensor, machine learning, behaviour

## Abstract

Previous research has shown that sensors monitoring lying behaviours and feeding can detect early signs of ill health in calves. There is evidence to suggest that monitoring change in a single behaviour might not be enough for disease prediction. In calves, multiple behaviours such as locomotor play, self-grooming, feeding and activity whilst lying are likely to be informative. However, these behaviours can occur rarely in the real world, which means simply counting behaviours based on the prediction of a classifier can lead to overestimation. Here, we equipped thirteen pre-weaned dairy calves with collar-mounted sensors and monitored their behaviour with video cameras. Behavioural observations were recorded and merged with sensor signals. Features were calculated for 1–10-s windows and an AdaBoost ensemble learning algorithm implemented to classify behaviours. Finally, we developed an adjusted count quantification algorithm to predict the prevalence of locomotor play behaviour on a test dataset with low true prevalence (0.27%). Our algorithm identified locomotor play (99.73% accuracy), self-grooming (98.18% accuracy), ruminating (94.47% accuracy), non-nutritive suckling (94.96% accuracy), nutritive suckling (96.44% accuracy), active lying (90.38% accuracy) and non-active lying (90.38% accuracy). Our results detail recommended sampling frequencies, feature selection and window size. The quantification estimates of locomotor play behaviour were highly correlated with the true prevalence (0.97; *p* < 0.001) with a total overestimation of 18.97%. This study is the first to implement machine learning approaches for multi-class behaviour identification as well as behaviour quantification in calves. This has potential to contribute towards new insights to evaluate the health and welfare in calves by use of wearable sensors.

## 1. Introduction

Our ability to assess animal behaviour is a key component of our ability to recognise ill-health and evaluate welfare in domestic livestock [1,2]. Deviations from normal behaviour can be reflective of pathology, an adaptive response to a health problem, a signal of vigour or of need [3]. However, the visual assessment of animal behaviour has numerous limitations such as the time, labour and expense needed to observe individual animals. New technologies such as wearable sensors and expert systems are transforming our ability to monitor animal behaviour, including that of livestock [4,5]. Behavioural data gathered by sensors can be continuously processed by expert systems capable of detecting abnormalities and warning the farmer where interventions are necessary [6]. In calves, sensors that monitor lying behaviour and step count in have been developed [7,8], recording data which can be useful in identifying early signs of ill health in calves [9,10]. However, the accuracies of sensors evaluating other behaviours such as rumination in calves are mixed [11] and the simultaneous identification of multiple behaviours requires further research. For example, one commercial sensor reasonably identified lying behaviours in calves compared to visual observations but failed to accurately identify feeding and drinking behaviours [12]. 

Monitoring a wider set of behaviours has been hypothesised to be of greater predictive value for detecting ill health in livestock than a more restricted set of behaviours [13,14]. In calves, behaviours such as activity whilst lying, self-grooming, feeding and locomotor play are likely to be informative for predicting health and welfare. For example, calves inoculated with bacterial lipopolysaccharides have been shown to decrease their time spent lying active and increase their time spent lying inactive, whilst total lying time was not affected [15]. Another behaviour of interest is self-grooming. Rats injected with an inflammatory cytokine show a dose dependant reduction in grooming behaviour [16] and grooming behaviour decreases in sick calves [10,15,17]. Alongside rumination and nutritive suckling (i.e., suckling milk at an automatic feeder), feeding behaviours monitored in calves could include non-nutritive suckling at the milk feeder. Non-nutritive visits to the milk feeder have been shown to decrease in sick calves prior to any reduction in overall feed intake [18]. Finally, changes in play behaviour could serve as an early indicator of ill health [19]. In calves, painful procedures such as disbudding as well as reduced feed allowance have been associated with reductions in locomotor play behaviour [20,21].

If an increase or decrease in specific behaviours identified by a sensor is to be used as an indicator of ill health in calves, this approach must be capable of estimating the distribution of each behaviour in an unlabelled dataset. This quantification task seems almost trivial and researchers have mostly either ignored it (as most studies present mainly a behaviour identification task) or have tried to solve it by simply counting the number of samples predicted as positive by the algorithm i.e., the Classify and Count Method [22]. However, such an approach fails to consider the fact that positive predictive value decrease with prevalence (i.e., [23]) and possible differences in behaviour prevalence between the training/test dataset and a new unlabelled dataset. This can result in vast overestimation of low prevalence behaviours. For example, an algorithm developed to identify play behaviour in calves overestimated occurrence by some 200% despite pre-processing to increase the prevalence of positive samples [24]. Overestimation has also been reported in other low-prevalence behaviours such as movement activity [25] and rumination [26]. Estimates were improved when the prevalence of these behaviours increased [26]. The importance and need for quantification methods has been discussed widely in human machine learning tasks [22] but, to our knowledge, no such methods have been presented in the precision livestock literature.

Finally, different behaviours may require different sampling frequencies and statistical features in order to be identified [27]. Sensor sampling rate and feature calculations significantly impact battery life and should therefore be optimised for the behaviours monitored and battery life required [27]. However, many studies fail to establish which signal features and sampling rate are most appropriate for the behaviours classified. 

In order to address the limitations outlined above we propose a novel approach to sensor-based behaviour monitoring in calves with the following aims:

1. Create machine learning algorithms to classify two postures (standing and lying) and seven behaviours (locomotor play, self-grooming, active lying, non-active lying, non-nutritive sucking at the automatic feeder, nutritive sucking at the feeder, and ruminating) using a single sensor.
2. Explore signal feature importance and the impact of sampling frequency on classification performance.
3. Implement a quantification algorithm to accurately estimate the number of samples of locomotor play behaviour in test dataset with a low prevalence of positive samples.

## 2. Materials and Methods

### 2.1. Raw Data Collection

Thirteen Holstein dairy calves were selected by random number generator from a pen of 20. The calves selected were between 5–7 weeks old and housed in a straw-bedded pen (6 m × 12 m) along with the 7 other (non-trial) calves. An automatic feeder (Forster Technik COMPACT smart) fed calves milk replacer based on an individualised feeding plan and calves had ad-lib access to concentrates, chopped straw and water. The study was conducted at the Centre for Dairy Science Innovation at the University of Nottingham, UK. Ethical permission was obtained for the School of Veterinary Medicine and Science, University of Nottingham (unique reference number 1481 150603). 

**Figure 1:**
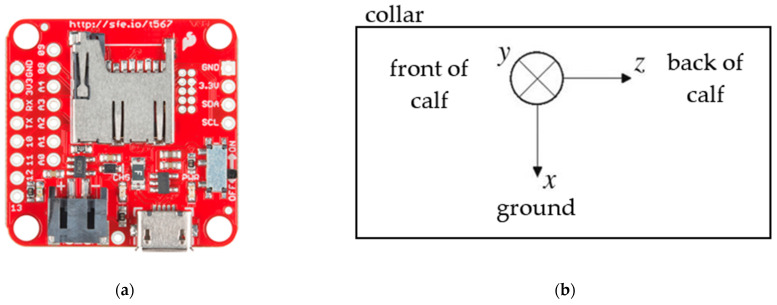
Photo of sensor used (**a**) sensor orientation on the collar (**b**).

The study duration was 12 days of which 2 days were used as a pilot for troubleshooting and preparation and 10 days for data collection. During week 1 (21/01/2019–25/01/2019) six calves were enrolled in the study. During week 2 (28/01/2019–02/02/2019) seven different calves were enrolled. Enrolled calves were caught daily by trained handlers. Each calf was equipped with a neck-worn collar onto which we had previously attached a sensor (Figure 1). The sensor was firstly placed in a lightweight plastic bag before being wrapped in tape and then attached to a collar using plastic cables and tape. Each sensor was fixed at the same orientation and location on the collar for consistency. 

Sensors recorded continuously from approximately 16.00 until 13.00 h the following day when they were removed and replaced with new sensors. The specific sensors used were SparkFun 9 degrees of freedom razor IMU MO sensors (www.sparkfun.com) which combine a SAMD21 microprocessor with an MPU-9250 9DoF sensor. The device was set to record data from a 3-axis accelerometer and a 3-axis gyroscope. Sampling rate was set to 100 Hz with a range of ±8 g and gyroscope range was 2000 °/s.

### 2.2. Behavioural Observations

Calf behaviour was recorded using four video cameras (5 Mp, 30 m IR. Hikvision Digital Technology Co., Ltd., Los Angeles, CA, USA). Three cameras were mounted on the walls of the pen at 3–4 m of height and the fourth was mounted on a tripod overlooking the automatic milk feeder. The cameras were oriented to ensure maximum cover of the pen. The cameras were set to record at high quality video (HEVC, H.265; and at 2944 × 1656 pixels′ quality) and 30 frames/s. Cameras were connected to a 4 MB video recorder (Hikvision Digital Co., Ltd., CA, Los Angeles, USA) from which data were retrieved using an external hard drive. 

Definitions for postures and behaviours for enrolled calves were recorded by three trained observers using the video recordings according the ethogram shown in Table 1. Precise time stamps (start and stop) of postures and behaviours were recorded manually [27]. A reliability test showed on average good to high agreement between observers (kappa > 0.7–0.9). Video footage was labelled between 16.00–20.00 h daily and only behaviours with a duration of more than 3 s were recorded. Each behaviour was labelled for a maximum of one hour per calf. The exception to this was locomotor play behaviour where all instances were labelled.

### 2.3. Data Processing

Merging of the behavioural observations and raw sensor data according to timestamp was performed using custom made scripts written in Python 3.5. Visualization of recorded accelerometer magnitude alongside the associated video recording was performed for each sensor recording in order to check any possible delays between video camera and sensor data due to sensor desynchronization. Any delays due to time stamp desynchronization were corrected. Data for the first four hours of sensor recording were used for the analysis.

Individual data files of both sensor and labelled data were discretised into windows of equal length. In this study windows sizes of 1 s–10 s with a 50% overlap [28], were explored. The set of feature characteristics was extracted from the magnitude of the acceleration and the magnitude of the gyroscope which are defined by $\bar{A}=\sqrt{A_{x}^{2}+A_{y}^{2}+A_{z}^{2}}$ and $\bar{G}=\sqrt{G_{x}^{2}+G_{y}^{2}+G_{z}^{2}}$, respectively, where *A_x_*, *A_y_*, *A_z_*, *G_x_*, *G_y_*, *G_z_* represent the acceleration and gyroscope signals at the axes *x*, *y*, *z*, respectively. Forty-four feature characteristics were computed using a previously defined set of features [27]. Full details of the definition and formula of the feature characteristics can be found in [27].

For the classification algorithm, the merged data contained all labelled behaviours and the sensor features (Dataset 1). For the quantification algorithm we merged sensor features characteristics from both labelled play and non-labelled play behaviour data (Dataset 2).

### 2.4. Classification Algorithm

An AdaBoost ensemble learning algorithm [29] was implemented using the fitcensemble function in Matlab 2019a. The AdaBoost algorithm learner was set to have a minimum leaf size of 5 and maximum number of splits per tree.

Classification performance for postures and activities was evaluated using a 5-fold cross validation which is a commonly used technique for robust evaluation of performance in classification [30]. Within this technique the original dataset was split into 5 subsets of equal size, and a total of 5 iterations are performed. At each iteration, 4 subsets are used to train the classification algorithm and the remaining one is held back to test. At each fold, performance values are computed using the test set and the average of these are used to represent the performance of the cross validation. As Dataset 1 was relatively well balanced for posture (i.e., standing and lying), there were 81,492 samples at 3 s for lying and 31,951 samples with 3-s (3s) windows for standing, no further processing was required.

However, for the different behaviours an under-sampling balancing technique was applied to address the problem of inter-class imbalance [31] i.e., behavioural classes not being equally represented due to the nature of the different activities. For example, locomotor play behaviour occurred rarely compared to non-active lying. The technique used the total number of samples of locomotor play behaviour as a measure to select samples for each individual behaviour across individual files. More precisely, let $\alpha_{i,b}^{im}$ represent the number of samples of behaviour b collected for individual dataset *i* over the original imbalanced dataset (*im*). Hence, the original imbalanced dataset $\left\{\alpha_{i,b}^{im}\right\}$ was balanced according to:

(1) αi,bbal={randomly selected a total of sminkbsamples from{αi,bim} if αi,bim>sminkb|αi,bim|   if     αi,bim≤sminkb

where $|\alpha_{i,b}^{im}|$ represents the cardinality (number of samples) of behaviour *b* collected from the individual datafile *i* in the original dataset and $k_{b}$ ($0<k_{b}\leq N$, where *N* is the total number of datafiles) that contain samples on behaviour *b* and $s_{min}$ is the minimum number of samples to balance the data. This method ensures balanced data for each individual across all the different behaviours, since the number of data samples per individual per class will be equal to or splaykb or $|\alpha_{i,b}^{im}|$. After balancing the data, the total number of 3-s window samples was 396 for active-lying, 396 for non-active lying, 396 for ruminating, 392 for non-nutritive suckling, 396 for nutritive suckling, 272 for self-grooming and 393 for locomotor play. Within this evaluation, performance was assessed using metrics which included overall accuracy, precision, recall, *F*-score and Cohen′s Kappa [32] as defined in [33].

### 2.5. Quantification Algorithm

An adjusted count (AC) method with a maximum selection threshold as described in (Forman 2008) was implemented using Dataset 2. All instances of locomotor play behaviour (P) were labelled for Dataset 2 and hence any sample that does not have a label can be consider as non-play (NP).

The AC algorithm is a two-step algorithm that corrects the estimate provided by a binary classifier using its true positive rate (*tpr*) and false positive rate (*fpr*). The AC algorithm first trains a binary classifier and then estimates *tpr* = true positives(*tp*)/(true positives(*tp*) +false negatives(*fn*)) and *fpr* = false positives(*fp*)/(true negative(*tn*) +false positives(*fp*)) by means of a cross-validation over the training set. In the second step, AC corrects the prevalence of an unknown sample using the following formula:

(2) $$p^{\prime }=\frac{p_{0}^{\prime }-fpr}{tpr-fpr}$$

where $p_{0}^{\prime }$ is the initial estimate of prevalence from the prediction of the classifier, $p^{\prime }$ is the adjusted prevalence and fpr and tpr are as previously described. When applied to highly imbalanced datasets, the performance of the AC method degrades quickly. Class imbalance can be solved by selecting a threshold that maximises tpr-fpr (denominator in the above formula) over a varying range of training conditions.

When applying the AC method for the quantification of play behaviour we first split Dataset 2 into two subsets of equal size: a training subset and a test subset. From the training subset a varying range of training conditions was generated by randomly selecting *p* = 10, 20, 30,…, 190 positive play instances and NP = 10,000 non-play instances for the training subset. The total number of positive play cases was 196 and negative cases was 70,983.

For each value of the varying range of training condition we estimated tpr and fpr characteristics via a 5-fold cross validation on a binary ensemble classifier algorithm for play and non-play. The threshold was selected using the training conditions that maximised tpr-fpr. Afterwards, initial training conditions that maximised the threshold were used to train a binary ensemble classification algorithm. This algorithm was then used to predict the number of play behaviour samples over the test subset. Predictions made by the classification algorithm were adjusted according to the above formula.

Since all instances of play behaviour were labelled in Dataset 2, it was possible to compare the adjusted number of window samples predicted as play with the total of samples observed as play behaviour via non-parametric correlation. Additionally, the total number of over or under estimation of instances of play behaviour were computed. Similarly, the number of instances of over/underestimation was computed for each individual data file.

### 2.6. Feature Ranking and Down-Sampling

Ranking of the 44 feature characteristics was obtained using ReliefF feature selection [34] for the classification of posture and behaviours.

We investigated the effect that different sampling frequencies can have on the performance of the classification. This was achieved by down-sampling the data originally sampled at 100Hz to sample frequencies of 50 Hz, 20 Hz, 10 Hz and 4 Hz. Down-sampling was performed by selecting a subset of the original raw dataset as follows:

${\left\{a_{2*i+1}\right\}}_{i=0}^{i=N/2}$ for down-sampling to 50 Hz.
${\left\{a_{5*i+1}\right\}}_{i=0}^{i=N/5}$ for down-sampling to 20 Hz.
${\left\{a_{10*i+1}\right\}}_{i=0}^{i=N/10}$ for down-sampling to 10 Hz.
${\left\{a_{25*i+1}\right\}}_{i=0}^{i=N/25}$ for down-sampling to 4 Hz.

where *N* is the total number of samples at 100 Hz. After data were down-sampled an ensemble classification algorithm for postures and behaviours was generated and assessed using a 5-fold cross validation in the same manner as previously described previously (Section 2.4).

## 3. Results

### 3.1. Classification Results

**Figure 2:**
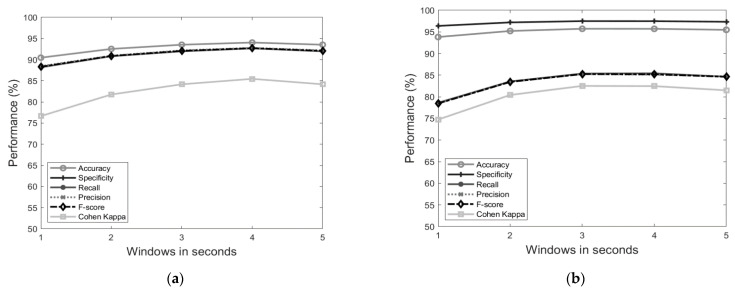
Performance (%) of the classifier for postures (**a**) and behaviours (**b**) as described in Table 1 across different window sizes (1–5 s). Metrics are computed as the mean of the postures/behaviours.

**Figure 3:**
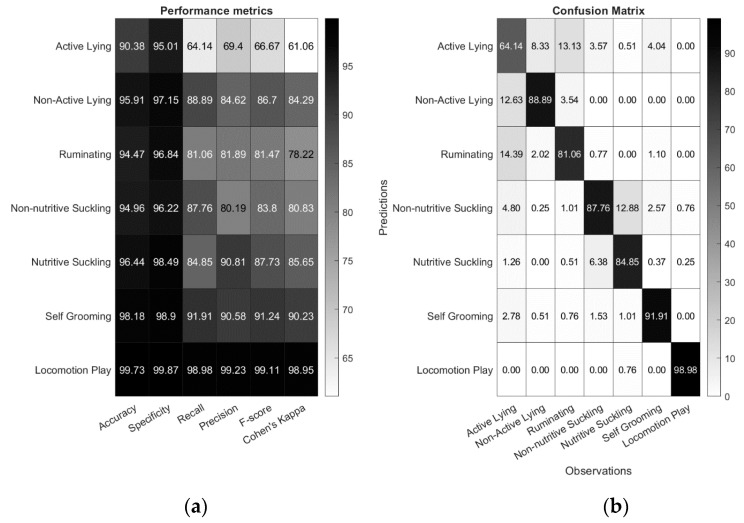
Classification performance metrics for behaviours (**a**) and confusion matrix (**b**) shown as a percentage, the darker the shading the higher the performance. Results shown in these figures were computed using a 3-s window size.

An initial comparison of the performance of the classification across windows sizes of 1–5 s was investigated for both posture and behaviours. The best results for posture were found when using a 4-s window (Figure 2) with an 94.38% overall accuracy, 92.99% specificity, 92.99% recall, 93.11% precision, 93.05% F-score and a Cohen’s Kappa of 0.8611. The best results for behaviour were using a window of 3s (Figure 2) providing an 95.72% overall accuracy, 97.46% specificity, 85.36% recall, 85.24% precision, 85.24% *F*-score and Cohen′s Kappa of 0.8247. Detailed results for classification performance and the confusion matrix using a 3s window for the behaviours are presented in Figure 3.

### 3.2. Feature Ranking and Down-Sampling

Results of the top 10 feature ranking are shown in Table 2.

**Figure 4:**
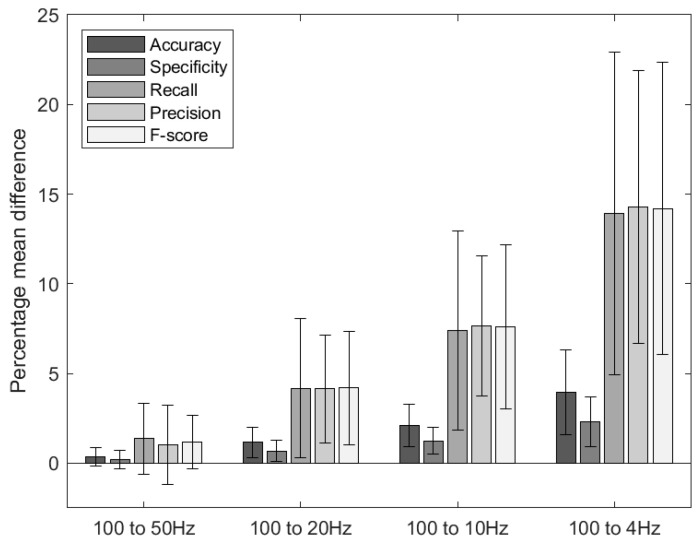
Decrease in the performance when down-sampling from 100 Hz to 50 Hz, from 100 Hz to 20 Hz, from 100 Hz to 10 Hz and from 100 Hz to 4 Hz. The bars show the average decrease across all the different behaviours, the black error bars show the variation in reduction (s.d) across the different behaviours.

The effect of down-sampling to the frequencies 50 Hz, 10 Hz, 10 Hz and 4 Hz is shown in Figure 4, which exhibits the average decrease in performance (accuracy, specificity, recall, precision and f-score).

A decrease in performance was obtained with a decreased sample frequency (i.e., a decrease of 0.34% (±0.50% s.d) in accuracy when down-sampling from 100 Hz to 50 Hz and a decrease of 3.94% (±2.34% s.d) in accuracy from 100 Hz to 4 Hz). However, the largest decreases were obtained in recall (13.92% ± 9% s.d), in precision (14.27% ± 7.62% s.d) and in *F*-score (14.18% ± 8.15% s.d) when down-sampled to 4 Hz.

The percentage decrease in performance when sampling is detailed by behaviour in Table 3. Play behaviour was the least affected by down-sampling (decrease in *F*-score by only 1% when down-sampled to 4 Hz), whilst performance statistics for active lying, ruminating, non-nutritive suckling, nutritive suckling and self-grooming were more severely affected (decrease in *F* score by 19.89%, 17.21%, 19.73%, 21.41% and 15.64%) when down-sampled from 100 Hz to 4 Hz. Non active lying was only moderately affected when down-sampling to 4 Hz (4.79% decrease in *F* score).

### 3.3. Quantification Results

**Figure 5:**
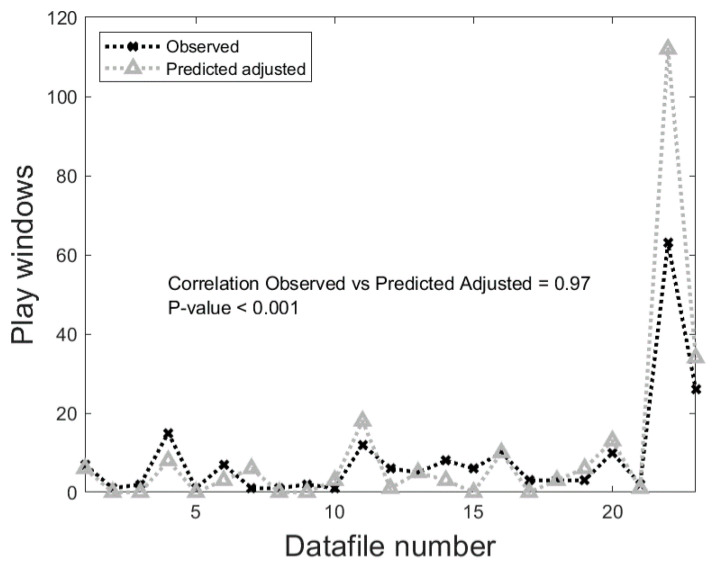
Comparison of the number of window samples predicted and the number of window samples observed for each individual dataset.

Quantification of play behaviour using Dataset 2 and the AC method is shown in Figure 5 where the number of window samples predicted as play vs. the number of observed play windows is shown. A significant positive correlation of 0.97 (*p*-value < 0.001) was obtained between observed and predicted play behaviour. The total number windows 72,377 of which 195 were play (0.27%) vs. 232 predicted, providing a total overestimation of 37 (18.97%).

## 4. Discussion

To the authors’ knowledge this the first study to develop an algorithm that can successfully identify such a diverse range of behaviours in calves using a sensor. In addition, this is also the first attempt in precision livestock research to develop and present a machine learning quantification algorithm. Our results demonstrate that signal data from a collar-based movement sensor can be used to accurately classify lying and standing posture whilst simultaneously identifying locomotor play, self-grooming, active lying, inactive lying and different feeding behaviours. Several of these behaviours have not been previously identified using a sensor such as self-grooming and non-nutritive suckling. Our algorithm also a reported high level of accuracy for most behaviours. Previous research using collar-based accelerometers has reported high levels of misclassification between feeding behaviours and posture in dairy cattle [35,36] and in sheep [37]. One explanation for the high performance of our algorithm is the inclusion of both gyroscope and accelerometer signal-based features, which were evenly ranked in the top ten features (Table 2). This finding reflects research in sheep showing that improved behavioural classification performance can be achieved when gyroscope features are included [27]. Active lying was the behaviour with the overall worst performance (90% accuracy, 64% sensitivity and 69% precision). It was more regularly confused with similar behaviours such as non-active lying and ruminating (Figure 3) Misclassification between different resting behaviours (i.e., lying awake and non-rapid eye movement sleep) has previously been reported in calves [38]. It is possible that the sensor may not be sufficiently sensitive to consistently detect the slight movements of active lying or differentiate active lying from ruminating. Additionally, a different behaviour was only assigned if it lasted longer than 3 s; it is therefore possible that short periods of non-movement during episodes of active lying were sufficient to allow misclassification as non-active lying. Another problem could be that the definition of active lying was broad (lying with head moving—see Table 1). Further differentiation of activities during active lying (i.e., chewing forage and social-grooming) could aid in reducing potential misclassification with similar behaviours (i.e., ruminating and self-grooming) and identify other interesting behaviours.

One behaviour of interest is locomotor play which was identified with 98.98% sensitivity, 99.73% specificity and 99.23% precision by our classification algorithm. Recent studies have explored the potential of commercially available leg worn sensors, to identify play behaviour in calves. One study used a summary acceleration data feature (motion index) to predict whether play was present or absent within predefined sampling periods (1 min or 15 min) [39]. The optimised threshold was an accurate predictor of whether play occurred or not in each 15 min. However, this approach does not allow metrics such as the behaviour′s duration and frequency to be calculated as multiple behaviours can occur within the same sampling period. Another approach has been to use raw accelerometer recordings of play behaviour in order to train and test a classifier [24]. Whilst the classifier’s predictions for play behaviour were correlated with observations (Pearson r = 0.87) predictions overestimated play occurrence by approximately 200% when predicting on a subset of the full dataset where locomotor play behaviour had low prevalence (6.5%). To address this problem, we implemented an adjusted count quantification algorithm on a binary classifier [22] using locomotor play behaviour as an example. Our quantification algorithm provided a high level of correlation with real observations (0.97; *p*-value < 0.001). Our results show promise since play behaviour was only overestimated by 19% despite its very low prevalence (0.27%). This is significant improvement from any published work in the field so far. Classifiers are not perfect and the test class distributions (i.e., behaviour distributions in real world) are not generally representative of the training dataset. We believe this offers a method for researchers to apply quantification for behavioural monitoring in livestock. In our current work we were limited by the complete labelled data being only available for locomotor play behaviour. To deliver a complete framework that can accurately monitor a larger number of low prevalence behaviours, a multi-class quantification algorithm needs to be implemented. This is included in our future work.

We should highlight that our neither our proposed quantification algorithm nor classification algorithm addresses changes that could occur due to “concept drift”, this is where the feature space may change and algorithm performance may be altered [40]. Concept drift occurs where a sensor which has been developed in a particular environment under-performs when that environment changes. In precision livestock this could be due to differences in the animals (age, breed, etc.) and environment characteristics (i.e., elevation, soil, particular farm constraints.) Our previous work has led to the development of algorithms to deal with possible concept drift [41]. Further studies could explore algorithm verification in new environments to confirm the accuracies reported.

Our study also explored which sampling frequencies were most appropriate for our classification algorithm. A small decrease in the performance metrics occurred when we down sampled to 50 Hz and a more significant decrease when down-sampling to 4 Hz (i.e., a 14.18% decrease in F-score) (Figure 4). Interestingly, there was minimal decrease in classification performance for locomotor play and non-active lying even when down-sampled to 4 Hz. This is likely due to the characteristic high amplitude acceleration pattern of locomotor play behaviour and the low amplitude pattern for non-active lying indicating that these can be differentiated from other behaviours even at lower sampling frequencies. Active lying, ruminating, self-grooming, nutritive suckling and non-nutritive suckling suffered more significant decreases in performance when down-sampled to 4 Hz. This indicates the necessity to identify more complex signal patterns in order to classify these behaviours. If sampled at a low frequency one approach to improve classification performance could be to increase window size and thereby increase the likelihood of identifying a characteristic signal pattern. Sampling between 20 Hz and 10 Hz is a good compromise between algorithm performance and battery life dependent on the intended application. This confirms previous findings using a similar sensor in sheep which recommended a sampling frequency of 16 Hz [27].

Finally, behaviours identified in this study were chosen for their relevance to calf health and welfare as well as for regular occurrence in pre-weaned calves and relative ease of labelling. Behaviours that could be incorporated by future studies include further drinking [42] and feeding behaviours [43] (i.e., drinking from water trough, eating roughage, eating concentrate) other resting behaviours (i.e., non-rapid and rapid eye movement sleep, non-active standing), walking, social grooming, stereotypical behaviours such as navel sucking as well as possible health indicators such as coughing or laboured breathing. Considerations include sampling frequency required, the need to carefully define behaviours, the labour requirements to label behaviours and the need to implement multi-class quantification methods for those behaviours that occur at low prevalence.

## 5. Conclusions

We have addressed two key challenges that are encountered by precision livestock research (a) develop a classification algorithm for a breadth of behaviours using a single sensor and (b) quantify the distribution of the behaviour in a real-world scenario where class distribution is different than the training set. Our classification algorithm was able to identify accurately a breadth of behaviours e.g., posture, maintenance behaviours such as self-grooming, feeding behaviours, resting behaviours and locomotor play. We demonstrated that sampling frequencies between 10 Hz and 20 Hz could be a reasonable trade-off between accuracy and computational power. Features from both the accelerometer and gyroscope are crucial for achieving high accuracies for classification. Furthermore, we developed and demonstrated accuracy of a quantification algorithm for predicting behaviour distribution; an area mostly ignored in precision livestock. Our results showed high accuracy with relatively low overestimation in unseen real-world data despite very low behaviour prevalence. This is of particular importance for research where change in behaviour distribution is of interest for disease and welfare prediction and quantification. Further work will involve further developing multiclass quantification algorithms and using the developed methods to improve our knowledge of the relationship between an individual′s behaviour, its health and its welfare.

## Figures and Tables

**Table 1:** Definition of the calf postures and behaviours used for the classification.

**Posture states**	**Description**
Lying	Calf is lying down on the sternum or side, body to the floor.
Standing	Calf is standing and shows head movements and may be moving one or more limbs in a forward or backwards motion.
**Behaviour states**	**Description**
Non-active lying	Calf lying down on the sternum or side, body on the floor with head not moving.
Active lying	Calf is lying down and with the head lifted from the ground, supported by the neck and moving.
Ruminating	Calf is lying down and shows regular jaw movements interrupted by regurgitation and swallow cycles with the head remaining in a constant position.
Self-grooming	All self-grooming movements where tongue is visible across body surface.
Nutritive suckling	Calf is standing in milk feeder, holds teat in his/her mouth and makes swallowing movements. The automatic feeder dispenses milk (milk flows through tube visible on video).
Non-nutritive suckling	Calf is standing in milk feeder, regularly (<every 3 s) holds teat in his/her mouth. The automatic feeder does not dispense any milk (milk does not flow through tube visible on video).
Locomotor play	Rapid forward movement that lasts 3 s or longer (in real time) and could include instances of jumping or bucking. It includes all instances of trotting (two beat leg movements synchronized diagonally), cantering (three-beat gait in between a trot and a gallop) and galloping (four-beat gait with a phase where all legs are off the ground).

**Table 2:** Top 10 ranked features using the ReliefF algorithm for both postures and behaviours. Italics have been used for gyroscope difference based features to differentiate them from acceleration difference based features. Frequency domain-based features can be differentiated from time domain features by the presence of an asterisk (*).

Postures		Behaviours
Rank	Feature Characteristics	Feature Characteristic
1	*Minimum*	*Difference Zero crossing*
2	First quantile *	*Zero Crossings*
3	Minimum *	*Kurtosis*
4	*Difference Kurtosis*	Difference Zero Crossing *
5	Difference Spectral Entropy *	Zero Crossing *
6	Mean	*Min*
7	Signal Area	Difference Spectral Entropy *
8	*Difference Zero Crossing*	Kurtosis
9	Difference Zero Crossing	*Difference Kurtosis*
10	Spectral Entropy *	Signal Area *

**Table 3:** Percentage decrease in algorithm performance by behaviour when down-sampling from 100 Hz to 50 Hz, from 100 Hz to 20 Hz, from 100 Hz to 10 Hz and from 100 Hz to 4 Hz.

Sampling Frequency (Hz)	50	20	10	4
	% decrease in Accuracy from 100 Hz			
Active Lying	1.33	2.16	3.37	4.78
Non-Active Lying	0.04	0.49	1.63	1.67
Ruminating	0.49	2.16	2.20	5.35
Non-nutritive Suckling	0.42	1.55	2.96	6.14
Nutritive Suckling	0.00	0.87	2.99	6.33
Self-Grooming	0.42	0.98	1.55	3.15
Locomotor Play	0.00	0.00	0.00	0.19
	% decrease in Specificity from 100 Hz			
Active Lying	1.34	0.67	0.89	1.74
Non-Active Lying	0.13	0.53	1.16	1.61
Ruminating	0.22	1.83	1.92	3.48
Non-nutritive Suckling	0.09	0.84	1.96	3.61
Nutritive Suckling	0.09	0.67	1.92	3.92
Self-Grooming	0.00	0.30	0.76	1.60
Locomotor Play	0.00	0.00	0.00	0.00
	% decrease in Recall from 100 Hz			
Active Lying	1.26	10.61	17.42	21.97
Non-Active Lying	1.01	0.25	4.29	2.02
Ruminating	2.02	4.04	3.79	15.91
Non-nutritive Suckling	2.30	5.61	8.67	20.66
Nutritive Suckling	0.00	2.02	9.09	19.95
Self-Grooming	4.78	6.99	8.52	16.73
Locomotor Play	0.00	0.00	0.00	0.25
	% decrease in Precision from 100 Hz			
Active Lying	5.72	6.86	11.04	16.88
Non-Active Lying	0.00	2.41	5.79	7.14
Ruminating	1.43	8.75	9.03	18.50
Non-nutritive Suckling	0.80	4.60	9.57	18.88
Nutritive Suckling	0.39	3.81	11.24	23.00
Self-Grooming	0.00	3.08	6.88	14.55
Locomotor Play	0.00	0.00	0.00	1.01
	% decrease in F-score from 100 Hz			
Active Lying	3.39	8.98	14.77	19.89
Non-Active Lying	0.24	1.40	5.09	4.79
Ruminating	1.73	6.44	6.47	17.21
Non-nutritive Suckling	1.49	5.07	9.19	19.73
Nutritive Suckling	0	2.86	10.11	21.41
Self-Grooming	2.31	5.05	7.69	15.64
Locomotor Play	0	0	0	0.63

## Data Availability

The data presented in this study are available on request from the corresponding author. The data are not publicly available due to privacy concerns as per industrial collaboration.
